# Quadriscupid Aortic Valve with Concurrent Aortic Stenosis and Insufficiency

**DOI:** 10.5334/jbr-btr.978

**Published:** 2016-02-08

**Authors:** Stephanie Vanden Bossche, Stephen Van Meerbeeck, Daniel Devos

**Affiliations:** 1 Universitair Ziekenhuis Gent, BE; 2 Radiologie Dokters Van Meerbeeck bvba, BE

**Keywords:** cardiac, magnetic resonance imaging, MRI, MR, quadricuspid aortic valve

## Abstract

We present the case of a 22-year-old man with a congenital mixed aortic valve dysfunction who underwent cardiac Magnetic Resonance Imaging (MRI) for the assessment of aortic valve morphology and function prior to valve replacement. Cardiac MRI showed a four-leaf-clover aortic valve morphology, the typical presentation of a quadricuspid aortic valve. The patient underwent a successful Bentall procedure to replace the aortic valve, aortic root and ascending aorta. This case report illustrates the MRI findings of a quadricuspid aortic valve with associated aortic stenosis and regurgitation.

## Introduction

A quadricuspid aortic valve (QAV) is a very rare congenital malformation that is often associated with valvular dysfunction, most likely aortic regurgitation, although mixed regurgitation and stenosis, and very rarely, isolated stenosis may also occur. The diagnosis can be made by transthoracic echocardiography (TTE), transoesephageal echocardiography (TOE), cardiac computed tomography (CT) and cardiac magnetic resonance imaging (MRI). In this paper we present the case of a 22-year-old man with a known congenital mixed aortic valve disease, in whom cardiac MRI revealed the presence of a quadricuspid aortic valve.

## Case presentation

A 22-year-old asymptomatic man presents at the cardiology department for follow-up of a congenital mixed aortic valve disease that presented first in childhood. Cardiac auscultation reveals a systolic decrescendo high-frequent ejection 2/6 murmur and diastolic 2/6 murmur. Transthoracic echocardiography shows a normotrophic mildly dilated left ventricle (end-diastolic diameter 63 mm) and a grade three aortic regurgitation combined with moderate aortic stenosis. The ascending aorta is also moderately dilated (48 mm). The left ventricular and ascending aorta dilatation appears to have advanced compared to previous sonographic measurements, indicating progressive regurgitation. Surgical intervention is advised and a preoperative cardiac MRI is planned.

**Figure 1:**
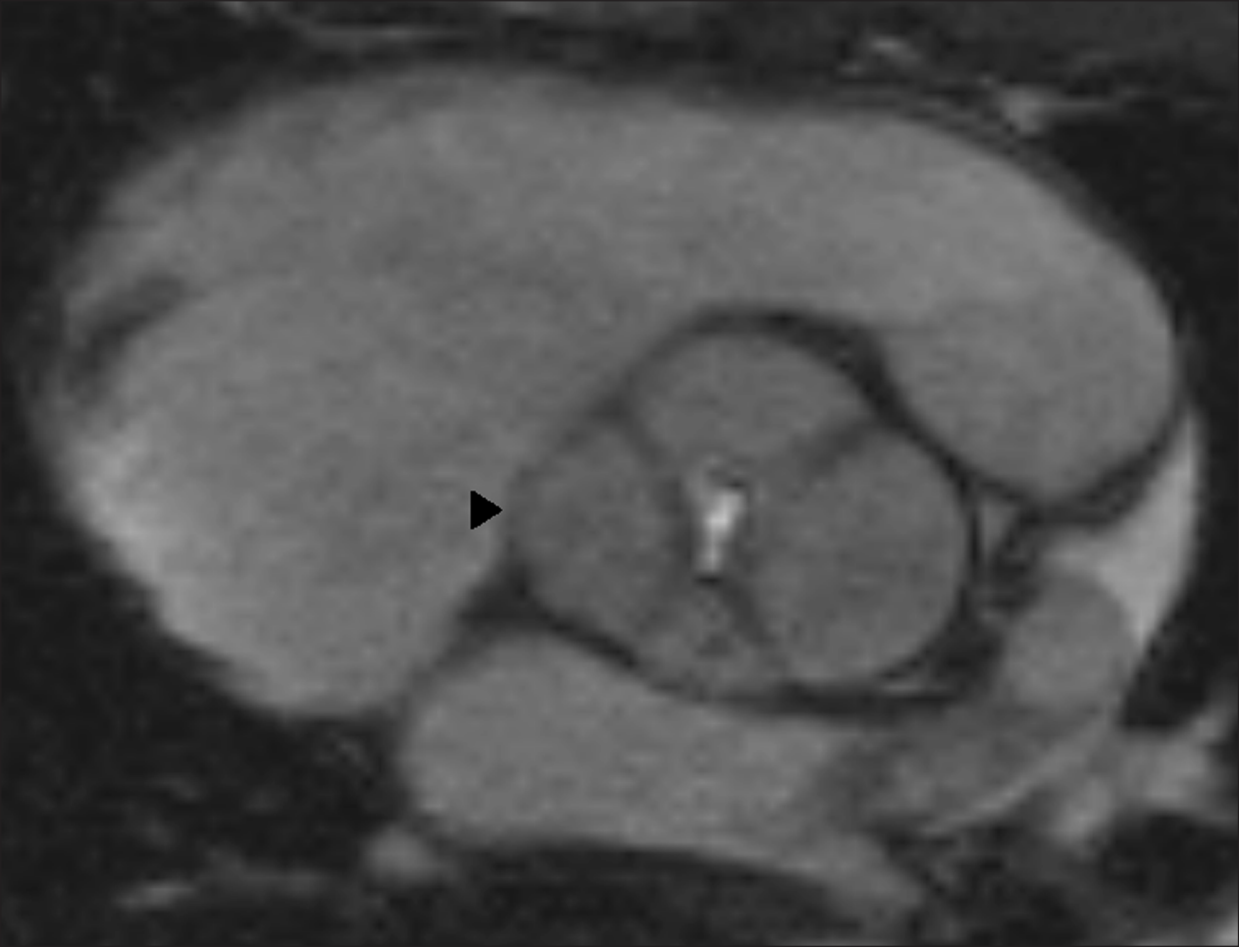
Cine MRI image in the aortic valve plane demonstrating the four-leaf-clover image (arrowhead) of the quadricuspid valve in diastolic phase. It is considered a type D QAV because there is one large cusp, two medium-sized cusps, and one smaller cusp.

**Figure 2A and B:**
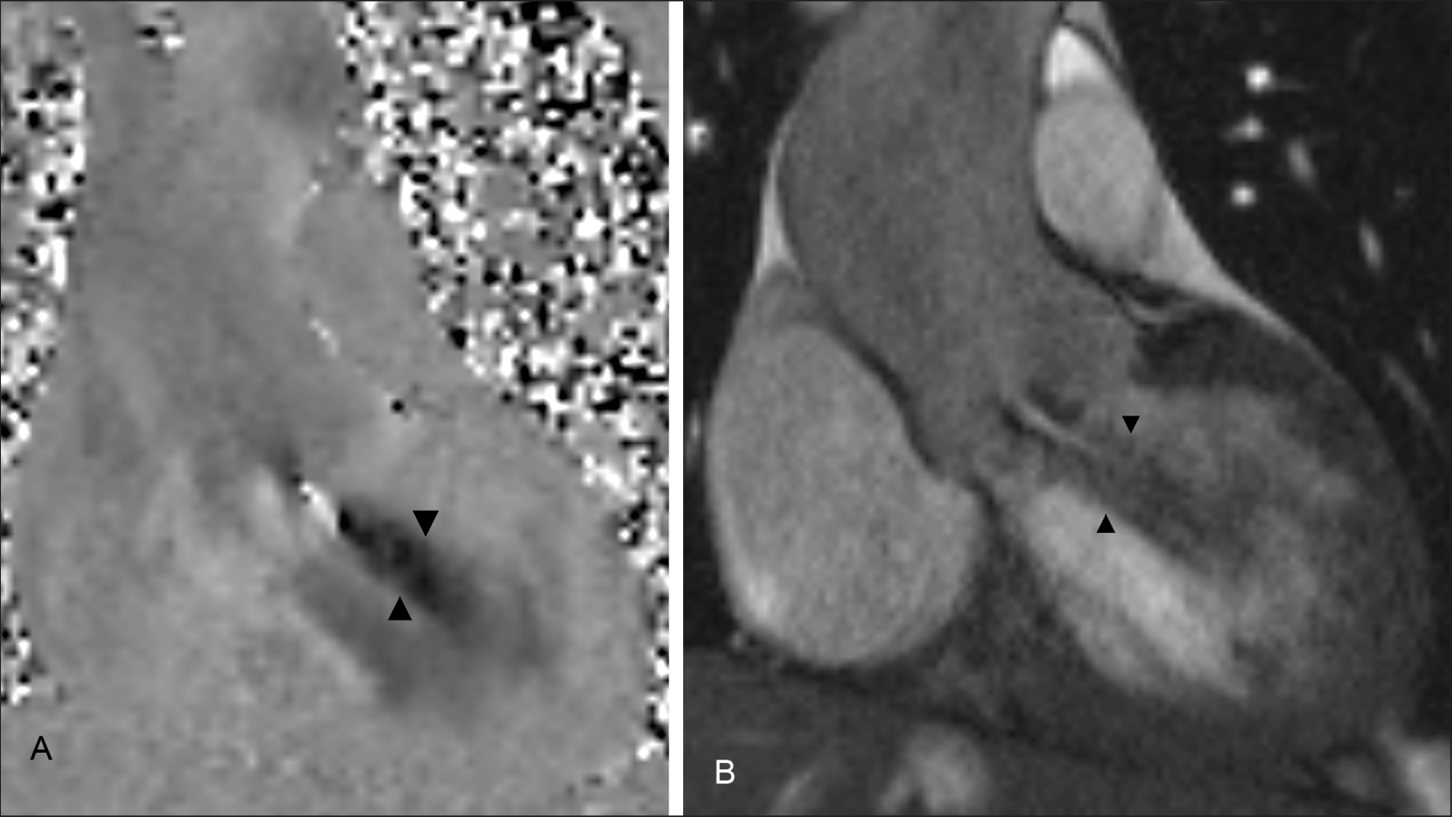
**(A)** Coronal flow velocity encoded images show holodiastolic regurgitating jet below the aortic valve (arrowheads), with a regurgitation fraction of 49% and regurgitant volume of 81 ml, indicating severe aortic insufficiency. **(B)** Coronal cine MRI images of the left ventricular outlet during diastole demonstrate the regurgitating jet (arrowheads) during diastole because of aortic insufficiency.

**Figure 3A and B:**
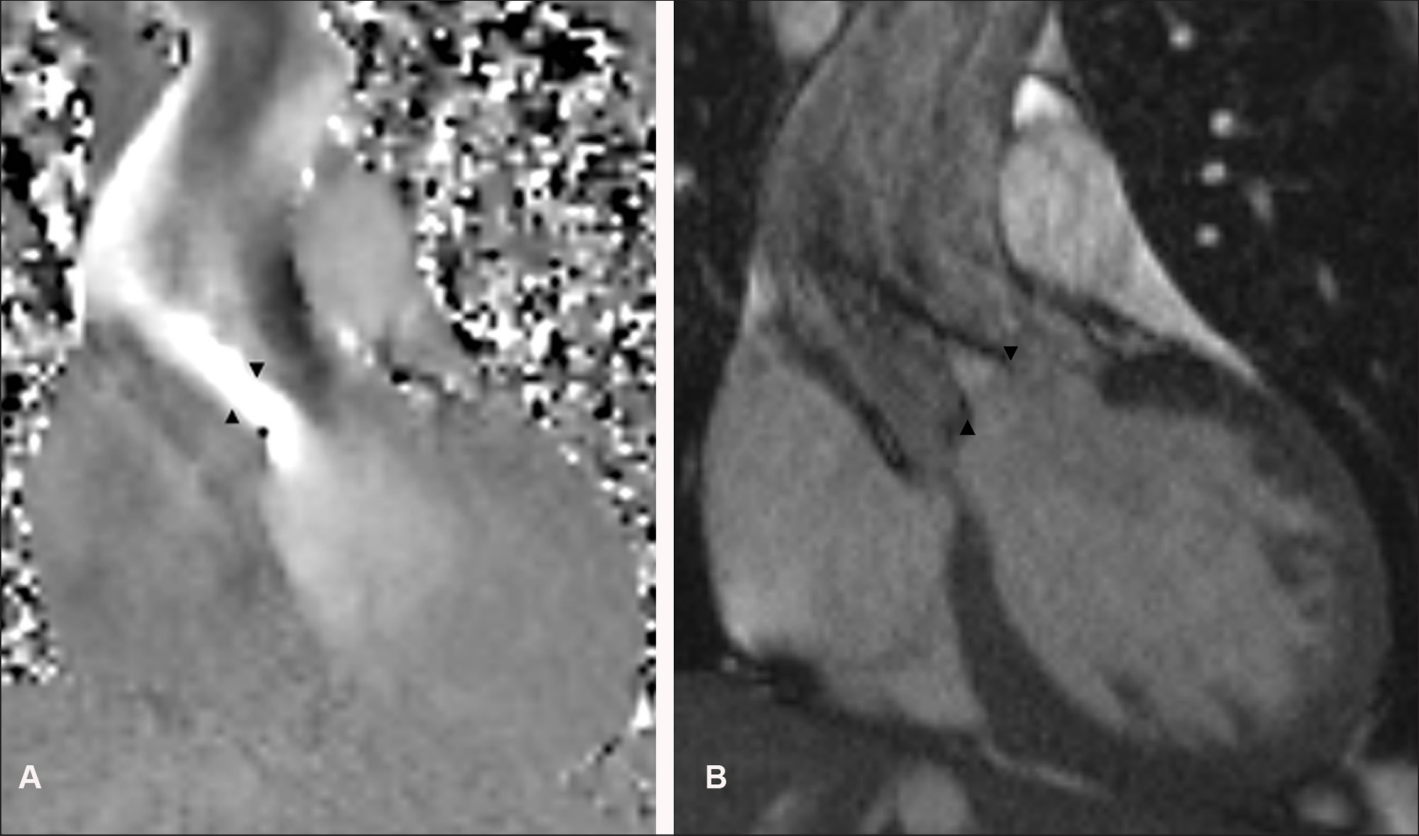
**(A)** Coronal flow velocity encoded images show high velocity blood flow across the aortic valve (arrowheads) compatible with a moderate aortic valve stenosis; a peak systolic velocity of 328 cm/s was measured. **(B)** Coronal cine MRI images of the left ventricular outlet during systole demonstrate a narrowed aortic valve orifice (arrowheads) during systole because of concurrent aortic stenosis.

On cine MRI images in the aortic valve plane, a four-leaf-clover aortic valve with one large cusp, two medium-sized cusps, and one smaller cusp is seen (Figure 1). Flow velocity encoded images and cine MRI show a marked holodiastolic regurgitating jet and early diastolic regurgitation in the ascending aorta. A regurgitation fraction of 49%, a regurgitating volume of 81 ml and a regurgitant orifice area of 0.5 cm^2^ are measured, indicating a severe aortic regurgitation (Figure 2). The high peak systolic velocity across the aortic valve, measured by flow velocity encoded imaging (328 cm/s) and a narrowed aortic valve orifice during systole, demonstrated by cine MRI confirm the aortic stenosis (Figure 3). The patient underwent a Bentall procedure. The aortic valve, aortic root and ascending aorta were replaced by a composite graft with re-implantation of the coronaries. Follow-up consultations up to one year after surgery show normal valve function and cardiac testing.

## Discussion

A quadricuspid aortic valve is a rare congenital variant, with an incidence of 0.008 at autopsy. There is a slight male predominance (1.6:1) and mean age at presentation is 51 years (range 2 to 84 years). A QAV is often associated with impaired aortic valvular function, most commonly aortic regurgitation (75%), less frequently a mixed pattern with presence of regurgitation and stenosis (8%) and rarely solitary aortic stenosis (1%). Valve function is normal in 16% of patients. Treatment options are follow-up, valve replacement or less frequently valve repair. In Tutarel’s series of 186 patients, 45% of patients required valvuloplasty [1].

The development of the aortic valve takes place in the fifth to ninth embryonic week. In week five, two mesenchymal ridges develop in the truncus arteriosus, fuse and descend in the ventricles to form the aorticopulmonary septum. At the junction of the bulbus cordis and truncus arteriosus three mesenchymal swellings form in the aortic and pulmonary ridges, which later form the tricuspid semilunar valves. The exact cause of QAV development is uncertain but some of the proposed mechanisms include anomalous conotruncal septation, excavation of one of the valve cushions and post-inflammatory valve cushion septation. In 10% of cases, QAV is associated with coronary artery variations, such as aberrant positioning of the coronary ostia or a single coronary artery. The concurrent coronary anomalies could be explained by the fact that coronary arteries develop shortly before the valves, so an early event might affect both [2].

The first case report diagnosed by imaging was written by Herman et al. in 1984. He described the pathognomonic X configuration—instead of the normal Y configuration of the aortic valve—, composed of the commissural lines of the closed QAV on transthoracic echocardiography [3]. On cardiac magnetic resonance imaging, the four-leaf-clover image in the aortic valve plane is typical for QAV [4]. Seven morphological types of quadricuspid valve have been described in 1973 by Hurwitz and Roberts, based on the size of each individual cusp: type A has four equal-sized cusps; type B has three equal-sized cusps and one smaller; type C has two larger and two smaller cusps; type D has one large cusp, two medium-sized cusps, and one smaller cusp; type E has three equal-sized cusps and one larger cusp; type F has two equal and two unequal smaller cusps; type G has four cusps of unequal size. Type B is the most common and type G the least common type [1]. The only differential diagnosis to be made is an acquired, pseudoquadricuspid aortic valve, which is a formerly normal cusp septated secondary to inflammatory damage in the setting of bacterial endocarditis [2].

## Conclusion

A quadricuspid aortic valve is a very rare congenital anomaly and possible cause of severe aortic regurgitation with sometimes concurrent stenosis. Diagnosis is possible with cardiac magnetic resonance by identifying the four-leaf-clover image in the aortic valve plane on cine MRI. Cardiac MRI can also be used in pre-operative planning to quantify the degree of aortic regurgitation and stenosis as well as to exclude associated cardiac abnormalities such as aberrant ostial positioning of the coronary arteries.

## Competing Interests

The authors declare that they have no competing interests.
